# Crystal structure of racemic (*R*/*S*,*E*)-2-(4-hy­droxy­phen­yl)-4-(2-phenyl­hydrazin-1-yl­idene)chromane-5,7-diol ethanol monosolvate

**DOI:** 10.1107/S2056989022002079

**Published:** 2022-03-01

**Authors:** Hemant P. Yennawar, Anna Sigmon

**Affiliations:** a Department of Biochemistry and Molecular Biology, 108 Althouse Laboratory, Pennsylvania State University, University Park, PA 16802, USA; b Pennsylvania State University, Brandywine Campus, 25 Yearsley Mill Rd., Media, PA 19063, USA

**Keywords:** disordered mixed enanti­omeric crystal structure, flavanone, hydrazone, naringenin

## Abstract

The crystal structure of racemic (*R*/*S*,*E*)-2-(4-hy­droxy­phen­yl)-4-(2-phenyl­hydrazin-1-yl­idene)chromane-5,7-diol ethanol monosolvate, C_21_H_18_N_2_O_4_·C_2_H_6_O, in a centrosymmetric lattice is reported. The two racemates occupy the same position in the asymmetric unit – *a disordered mixed enanti­omeric structure*. Hydrogen bonds of the type O—H⋯C(π) in addition to typical C—H⋯O, O—H⋯O and O—H⋯N are identified. A positional disorder is seen in the solvent mol­ecule (ethanol) as well. The phenyl­hydrazone group is nearly coplanar with the chromane ring system [dihedral angle = 15.5 (1)°], while the the 4-hy­droxy­phenyl ring is perpendicular [dihedral angle = 87.2 (1)°] to the chromane. The pyran ring has an envelope pucker [*Q* = 0.363 (3) Å, θ = 57.6 (3)°; and for the enanti­omer: *Q* = 0.364 (3) Å, θ = 127.4 (4)°].

## Abstract 2

The structure of racemic (*R*/*S*,*E*)-2-(4-hy­droxy­phen­yl)-4-(2-phenyl­hydrazin-1-yl­idene)chromane-5,7-diol ethanol monosolvate, has been determined. The packing is assisted by C—H⋯O, O—H⋯O, O—H⋯N and O—H⋯C(π) type hydrogen bonds, and the pyran ring has an envelope pucker.

## Chemical context

Naringenin is a naturally occurring flavanone compound found in citrus fruits, bergamot and tomatoes (Cai *et al.*, 2004 ▸). It has been reported to have a wide range of biological activities, including anti-viral, anti-inflammatory and anti-aging properties (Heim *et al.*, 2002 ▸). Due to its inherent medicinal properties, derivatives of naringenin have also been synthesized and studied as potential treatments for disease. The title compound, (*R*/*S*,*E*)-2-(4-hy­droxy­phen­yl)-4-(2-phenyl­hydrazineyl­idene)chromane-5,7-diol, is a hydrazone naringenin derivative that has been reported to induce apoptosis in human cervical cancer cells (Kim *et al.*, 2012 ▸). Its close structural analog, 5-hy­droxy-7,4′-di­acetyl­oxyflavanone-*N*-phenyl­hydrazone, exhibits cytotoxicity against non-small-cell lung cancer cells (Bak *et al.*, 2011 ▸). Despite their biological value, crystal structures have not been reported to date of any hydrazone derivatives of naringenin. Herein, we report the first crystal structure of a hydrazone derivative of naringenin. 
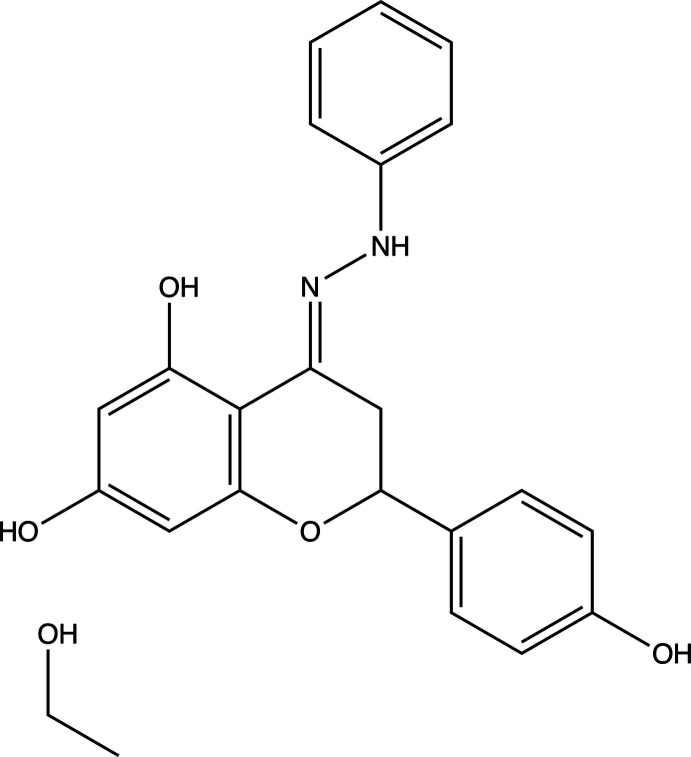


### Structural commentary

**Figure 1:**
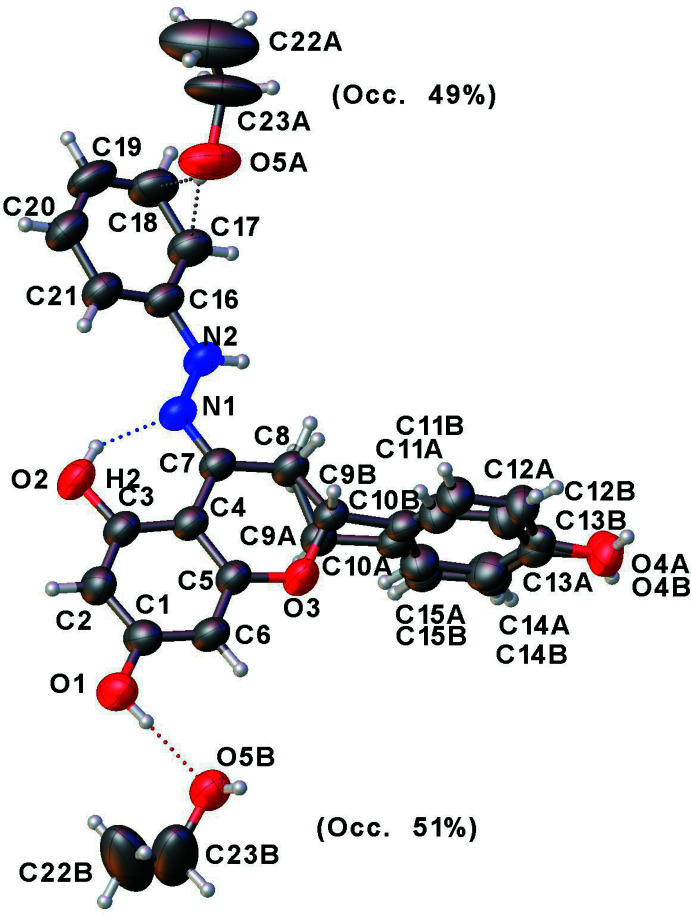
Displacement ellipsoid drawing at 50% probability level of the asymmetric unit showing the superposition of two enanti­omers in the asymmetric unit. The disorder in the solvent (ethanol) mol­ecule is resolved here, shown in two partial-occupancy locations.

The title compound along with the solvent (ethanol) mol­ecule in 1:1 ratio, yielded a disordered mixed enanti­omeric crystal in a centrosymmetric lattice (*P* 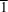 , Fig. 1 ▸). The structure was solved and refined in *P*1 and a distorted structure was found. The asymmetric unit has two racemates occupying the same position in a ratio of 0.562 (6):0.438 (6). Enanti­omeric structures in centrosymmetric lattices have been discussed by Flack (2003 ▸). The title mol­ecule has three phenyl rings, one of which is fused with a pyran ring. The mol­ecule in the asymmetric unit is a superposition of the two enanti­omers in the ratio of 0.562 (6):0.438 (6). The phenyl­hydrazone group is nearly coplanar with the chromane ring system [dihedral angle = 15.5 (1)°], while the the 4-hy­droxy­phenyl ring is perpendicular [dihedral angle = 87.2 (1)°] to the chromane. The pyran ring has an envelope pucker [*Q* = 0.363 (3) Å, θ = 57.6 (3)°; and for the enanti­omer: *Q* = 0.364 (3) Å, θ = 127.4 (4)°]. An intra­molecular O—H⋯N hydrogen bond exists between one of the hy­droxy groups on the chromane ring and the nitro­gen of the hydrazone group (Table 1 ▸). The carbon–nitro­gen double bond [N1=C7 = 1.295 (2) Å] exists as the *E* isomer.

### Supra­molecular features

**Figure 2:**
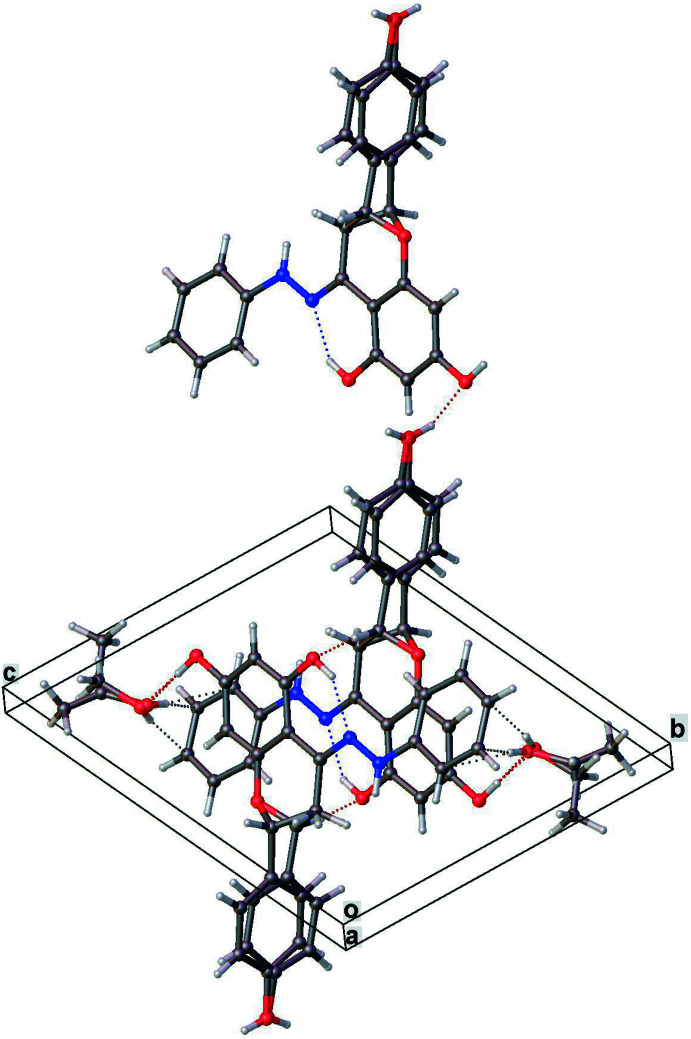
Crystal packing diagram showing intra­molecular O—H⋯N and inter­molecular O—H⋯O, C—H⋯O and (O—H⋯C(π) hydrogen bonds, as well as extensive π–π stacking inter­actions.

In the crystal, O—H⋯C(π) type hydrogen-bond inter­actions between the solvent ethanol and phenyl ring are observed (Table 1 ▸, Fig. 2 ▸). The phenyl ring is expected to have a partial negative charge because of the two nitro­gen atoms (known electron-releasing groups) just before the phenyl ring (Stewart, 1985 ▸). A database analysis of such inter­actions was reported by Viswamitra *et al.* (1993 ▸). The structure also has the not-so-rare C—H⋯O, O—H⋯O and O—H⋯N type hydrogen bonds. Extensive π–π stacking inter­actions [centroid–centroid distances in the range 4.223 (7) to 4.599 (5) Å] along the [1 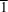 1] direction between the planar cores of neighboring mol­ecules further stabilize the lattice (Fig. 2 ▸).

## Database survey

A structure search was performed in Scifinder and Reaxys. A text search (‘flavanone’ and ‘chroman-4-yl­idene’ and ‘di­hydro­chromen-4-phenyl­hydrazone’) was performed in the Cambridge Structural Database (Groom *et al.*, 2016 ▸; accessed January 2022). To date, no crystal structures have been reported for a hydrazone derivative of naringenin, including the two flavanones mentioned in the *Chemical context* section. The most similar structures for which crystal data have been reported include acyl hydrazone derivatives of 2-phenyl­chroman-4-one and hesperetin. In particular, crystal structures for 2′-[2-(4-fluoro­phen­yl)chroman-4-yl­idene]isonicotino­hydra­zide (Nie *et al.*, 2006 ▸) and *N*-{(±)-[5,7-dihy­droxy-2-(3-hy­droxy-4-meth­oxy­phen­yl)chroman-4-yl­idene]amino}­benz­a­mide (Lodyga-Chruscinska *et al.*, 2015 ▸) have been reported.

## Synthesis and crystallization

The title compound was synthesized according to a previously reported procedure (Bak *et al.*, 2011 ▸).

## Refinement

Crystal data, data collection and structure refinement details are summarized in Table 2 ▸. The superposition of two enanti­omers in the asymmetric unit, and the disorder in the solvent (ethanol mol­ecule) necessitated 183 constraints. The hydrogen atoms were placed in their geometrically calculated positions and their coordinates refined using the riding model with parent-atom—H lengths of 0.93 Å (CH), 0.98 Å (chiral-CH), 0.96 Å (CH_3_), 0.97 Å (CH_2_), 0.86 Å (NH) or 0.82 Å (OH). Isotropic displacement parameters for these atoms were set to 1.2 (CH, NH) or 1.5 (CH_3_, OH) times *U*_eq_ of the parent atom. Idealized Me of the ethanol mol­ecule were refined as rotating group(s): C22*A* and C22*B* (H22*A* through *F*) and its idealized tetra­hedral OH refined as a rotating group: O5*A* and O5*B* (H5*A*, H5*B*).

## Supplementary Material

Crystal structure: contains datablock(s) I. DOI: 10.1107/S2056989022002079/jy2016sup1.cif

Supporting information file. DOI: 10.1107/S2056989022002079/jy2016Isup3.mol

Supporting information file. DOI: 10.1107/S2056989022002079/jy2016Isup3.cml

CCDC reference: 2153764

Additional supporting information:  crystallographic information; 3D view; checkCIF report

## Figures and Tables

**Table 1:** Hydrogen-bond geometry (Å, °)

*D*—H⋯*A*	*D*—H	H⋯*A*	*D*⋯*A*	*D*—H⋯*A*
O5*A*—H5*A*⋯C17	0.82	2.56	3.363 (15)	166
O5*A*—H5*A*⋯C18	0.82	2.47	3.263 (16)	162
O5*B*—H5*B*⋯C19^i^	0.82	2.59	3.405 (11)	173
O1—H1⋯O5*A* ^i^	0.82	1.79	2.590 (12)	166
O1—H1⋯O5*B*	0.82	1.90	2.709 (8)	170
C8—H8*BC*⋯O4*B* ^ii^	0.97	2.49	3.440 (17)	168
O4*A*—H4*A*⋯O1^iii^	0.82	1.89	2.677 (13)	160
C9*B*—H9*B*⋯O2^i^	0.98	2.39	3.347 (5)	165
O2—H2⋯N1	0.82	1.87	2.5975 (18)	147

Symmetry codes: (i) 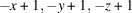 ; (ii) 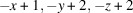 ; (iii) 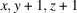 .

**Table 2:** Experimental details

Crystal data	
Chemical formula	C_21_H_18_N_2_O_4_·C_2_H_6_O
*M*_r_	408.44
Crystal system, space group	Triclinic, *P* 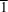
Temperature (K)	293
*a*, *b*, *c* (Å)	9.4329 (3), 10.9974 (4), 11.9310 (3)
α, β, γ (°)	115.244 (3), 93.939 (2), 104.180 (3)
*V* (Å^3^)	1064.01 (6)
*Z*	2
Radiation type	Cu *K*α
μ (mm^−1^)	0.74
Crystal size (mm)	0.2 × 0.19 × 0.13

Data collection	
Diffractometer	Rigaku Oxford Diffraction, Synergy Custom system, HyPix-Arc 150
Absorption correction	Gaussian (*CrysAlis PRO*; Rigaku OD, 2021 ▸)
*T*_min_, *T*_max_	0.638, 1.000
No. of measured, independent and observed [*I* > 2σ(*I*)] reflections	14477, 4067, 3077
*R*_int_	0.020
(sin θ/λ)_max_ (Å^−1^)	0.624

Refinement	
*R*[*F* ^2^ > 2σ(*F* ^2^)], *wR*(*F* ^2^), *S*	0.049, 0.161, 1.07
No. of reflections	4067
No. of parameters	381
No. of restraints	183
H-atom treatment	H-atom parameters constrained
Δρ_max_, Δρ_min_ (e Å^−3^)	0.24, −0.17

Computer programs: *CrysAlis PRO* (Rigaku OD, 2021 ▸), *SHELXT2018/2* (Sheldrick, 2015*a* ▸), *SHELXL2018/3* (Sheldrick, 2015*b* ▸), and *OLEX2* (Dolomanov *et al.*, 2009 ▸).

## supplementary crystallographic information

### Crystal data

**Table:**

C_21_H_18_N_2_O_4_·C_2_H_6_O	*Z* = 2
*M**_r_* = 408.44	*F*(000) = 432
Triclinic, *P*1	*D*_x_ = 1.275 Mg m^−^^3^
*a* = 9.4329 (3) Å	Cu *K*α radiation, λ = 1.54184 Å
*b* = 10.9974 (4) Å	Cell parameters from 8481 reflections
*c* = 11.9310 (3) Å	θ = 4.1–73.4°
α = 115.244 (3)°	µ = 0.74 mm^−^^1^
β = 93.939 (2)°	*T* = 293 K
γ = 104.180 (3)°	Block, yellow
*V* = 1064.01 (6) Å^3^	0.2 × 0.19 × 0.13 mm

### Data collection

**Table:**

Rigaku Oxford Diffraction, Synergy Custom system, HyPix-Arc 150 diffractometer	4067 independent reflections
Radiation source: Rotating-anode X-ray tube, Rigaku (Cu) X-ray Source	3077 reflections with *I* > 2σ(*I*)
Mirror monochromator	*R*_int_ = 0.020
Detector resolution: 10.0000 pixels mm^-1^	θ_max_ = 74.0°, θ_min_ = 4.2°
ω scans	*h* = −11→11
Absorption correction: gaussian (CrysAlisPro; Rigaku OD, 2021)	*k* = −13→13
*T*_min_ = 0.638, *T*_max_ = 1.000	*l* = −12→14
14477 measured reflections	

### Refinement

**Table:**

Refinement on *F*^2^	Hydrogen site location: inferred from neighbouring sites
Least-squares matrix: full	H-atom parameters constrained
*R*[*F*^2^ > 2σ(*F*^2^)] = 0.049	*w* = 1/[σ^2^(*F*_o_^2^) + (0.0996*P*)^2^ + 0.0482*P*] where *P* = (*F*_o_^2^ + 2*F*_c_^2^)/3
*wR*(*F*^2^) = 0.161	(Δ/σ)_max_ < 0.001
*S* = 1.07	Δρ_max_ = 0.24 e Å^−^^3^
4067 reflections	Δρ_min_ = −0.17 e Å^−^^3^
381 parameters	Extinction correction: *SHELXL2018/3* (Sheldrick 2015b), Fc^*^=kFc[1+0.001xFc^2^λ^3^/sin(2θ)]^-1/4^
183 restraints	Extinction coefficient: 0.0070 (13)
Primary atom site location: dual	

### Special details

**Table:**

Geometry. All esds (except the esd in the dihedral angle between two l.s. planes) are estimated using the full covariance matrix. The cell esds are taken into account individually in the estimation of esds in distances, angles and torsion angles; correlations between esds in cell parameters are only used when they are defined by crystal symmetry. An approximate (isotropic) treatment of cell esds is used for estimating esds involving l.s. planes.
Refinement. In light of the crystal structure with two enantiomer molecules sharing the same site in the asymmetric unit of P1, we tried refining the structure non-centrosymmetric P1 space-group, and saw the disorder in the chiral carbon persist even there, in both the independent molecules.

### Fractional atomic coordinates and isotropic or equivalent isotropic displacement parameters (Å^2^)

**Table:**

	*x*	*y*	*z*	*U*_iso_*/*U*_eq_	Occ. (<1)
O1	0.63930 (15)	0.57227 (12)	0.15536 (11)	0.0762 (4)	
H1	0.702063	0.648464	0.176530	0.114*	
O2	0.23838 (15)	0.35612 (12)	0.28729 (11)	0.0818 (4)	
H2	0.203745	0.365438	0.350612	0.123*	
O3	0.56642 (13)	0.82028 (11)	0.57204 (10)	0.0708 (4)	
N1	0.20641 (15)	0.48554 (13)	0.51936 (12)	0.0602 (3)	
N2	0.12754 (15)	0.49326 (14)	0.61217 (12)	0.0680 (4)	
H2A	0.142115	0.572868	0.677760	0.082*	
C1	0.56086 (19)	0.58198 (16)	0.24987 (14)	0.0620 (4)	
C2	0.43809 (19)	0.46783 (16)	0.22400 (15)	0.0661 (4)	
H2B	0.410135	0.389868	0.144550	0.079*	
C3	0.35768 (19)	0.47058 (16)	0.31680 (15)	0.0624 (4)	
C4	0.39633 (17)	0.58758 (15)	0.43746 (14)	0.0549 (4)	
C5	0.52020 (17)	0.70112 (15)	0.45827 (14)	0.0566 (4)	
C6	0.60281 (18)	0.69960 (15)	0.36667 (14)	0.0625 (4)	
H6	0.684912	0.776159	0.383312	0.075*	
C7	0.31398 (16)	0.59550 (15)	0.53822 (14)	0.0556 (4)	
C8	0.36188 (19)	0.73182 (16)	0.65742 (15)	0.0638 (4)	
H8A	0.273783	0.754978	0.686079	0.077*	0.562 (6)
H8B	0.415896	0.719516	0.722028	0.077*	0.562 (6)
H8BC	0.346340	0.712882	0.728678	0.077*	0.438 (6)
H8BD	0.300754	0.789928	0.654184	0.077*	0.438 (6)
C16	0.02353 (17)	0.37295 (17)	0.60136 (15)	0.0623 (4)	
C17	−0.0611 (2)	0.3883 (2)	0.69450 (18)	0.0748 (5)	
H17	−0.046879	0.476957	0.761543	0.090*	
C18	−0.1667 (2)	0.2717 (2)	0.6877 (2)	0.0878 (6)	
H18	−0.223327	0.282924	0.750162	0.105*	
C19	−0.1889 (2)	0.1394 (2)	0.5899 (2)	0.0914 (6)	
H19	−0.259464	0.061386	0.586085	0.110*	
C20	−0.1055 (2)	0.1245 (2)	0.4981 (2)	0.0850 (6)	
H20	−0.120445	0.035349	0.431597	0.102*	
C21	0.0010 (2)	0.23960 (18)	0.50212 (17)	0.0721 (5)	
H21	0.056675	0.227415	0.438926	0.087*	
O4A	0.7311 (17)	1.3456 (9)	1.1039 (9)	0.081 (3)	0.562 (6)
H4A	0.703178	1.409645	1.101761	0.121*	0.562 (6)
C9A	0.4577 (4)	0.8518 (3)	0.6438 (3)	0.0590 (10)	0.562 (6)
H9A	0.391142	0.878767	0.598130	0.071*	0.562 (6)
C10A	0.5317 (5)	0.9817 (5)	0.7668 (4)	0.0543 (10)	0.562 (6)
C11A	0.6354 (6)	0.9803 (5)	0.8543 (5)	0.0658 (12)	0.562 (6)
H11A	0.660538	0.898387	0.837484	0.079*	0.562 (6)
C12A	0.7007 (13)	1.1010 (7)	0.9660 (7)	0.0732 (17)	0.562 (6)
H12A	0.766711	1.098188	1.025843	0.088*	0.562 (6)
C13A	0.671 (2)	1.2248 (10)	0.9914 (13)	0.0566 (18)	0.562 (6)
C14A	0.5663 (11)	1.2260 (6)	0.9061 (7)	0.0598 (13)	0.562 (6)
H14A	0.541170	1.308011	0.923497	0.072*	0.562 (6)
C15A	0.4986 (6)	1.1050 (5)	0.7947 (5)	0.0599 (11)	0.562 (6)
H15A	0.428862	1.107069	0.737042	0.072*	0.562 (6)
O4B	0.751 (2)	1.3415 (10)	1.1041 (12)	0.0660 (19)	0.438 (6)
H4B	0.811016	1.329869	1.148770	0.099*	0.438 (6)
C9B	0.5225 (5)	0.8106 (4)	0.6767 (3)	0.0556 (12)	0.438 (6)
H9B	0.579037	0.755148	0.694866	0.067*	0.438 (6)
C10B	0.5752 (7)	0.9547 (5)	0.7898 (5)	0.0511 (12)	0.438 (6)
C11B	0.6689 (9)	0.9752 (6)	0.8945 (6)	0.0663 (15)	0.438 (6)
H11B	0.696726	0.899741	0.894502	0.080*	0.438 (6)
C12B	0.7214 (16)	1.1043 (8)	0.9980 (8)	0.0658 (18)	0.438 (6)
H12B	0.788594	1.117299	1.065956	0.079*	0.438 (6)
C13B	0.676 (3)	1.2142 (13)	1.0021 (16)	0.061 (2)	0.438 (6)
C14B	0.5818 (16)	1.1960 (9)	0.8993 (10)	0.070 (2)	0.438 (6)
H14B	0.552914	1.271569	0.900630	0.084*	0.438 (6)
C15B	0.5302 (8)	1.0657 (7)	0.7936 (6)	0.0631 (15)	0.438 (6)
H15B	0.464655	1.053270	0.724979	0.076*	0.438 (6)
O5A	0.1303 (15)	0.2093 (13)	0.7784 (9)	0.115 (3)	0.487 (7)
H5A	0.070539	0.240431	0.754421	0.172*	0.487 (7)
C22A	0.0763 (14)	0.0563 (13)	0.8743 (13)	0.225 (5)	0.487 (7)
H22A	0.138147	0.010641	0.820731	0.338*	0.487 (7)
H22B	−0.022803	−0.007894	0.850552	0.338*	0.487 (7)
H22C	0.116684	0.084083	0.960680	0.338*	0.487 (7)
C23A	0.0723 (17)	0.1585 (15)	0.8628 (12)	0.169 (4)	0.487 (7)
H23A	0.115468	0.236841	0.946343	0.203*	0.487 (7)
H23B	−0.032950	0.150256	0.849370	0.203*	0.487 (7)
O5B	0.8710 (12)	0.8064 (8)	0.2186 (6)	0.0826 (19)	0.513 (7)
H5B	0.951087	0.826239	0.263805	0.124*	0.513 (7)
C22B	0.9159 (9)	0.7123 (10)	0.0163 (6)	0.188 (4)	0.513 (7)
H22D	1.006501	0.697937	0.042231	0.282*	0.513 (7)
H22E	0.922411	0.727388	−0.056931	0.282*	0.513 (7)
H22F	0.833254	0.630478	−0.003716	0.282*	0.513 (7)
C23B	0.8974 (16)	0.8167 (14)	0.1032 (9)	0.170 (5)	0.513 (7)
H23C	0.813280	0.836918	0.071298	0.204*	0.513 (7)
H23D	0.984834	0.896724	0.125438	0.204*	0.513 (7)

### Atomic displacement parameters (Å^2^)

**Table:**

	*U*^11^	*U*^22^	*U*^33^	*U*^12^	*U*^13^	*U*^23^
O1	0.0905 (9)	0.0628 (7)	0.0673 (7)	0.0139 (6)	0.0337 (6)	0.0250 (5)
O2	0.0838 (8)	0.0538 (6)	0.0756 (7)	−0.0089 (6)	0.0211 (6)	0.0162 (5)
O3	0.0715 (7)	0.0538 (6)	0.0615 (6)	−0.0053 (5)	0.0229 (5)	0.0151 (5)
N1	0.0566 (7)	0.0533 (7)	0.0646 (7)	0.0045 (5)	0.0143 (6)	0.0278 (6)
N2	0.0672 (8)	0.0553 (7)	0.0673 (8)	−0.0019 (6)	0.0197 (6)	0.0255 (6)
C1	0.0715 (10)	0.0543 (8)	0.0617 (9)	0.0184 (7)	0.0221 (7)	0.0270 (7)
C2	0.0749 (10)	0.0510 (8)	0.0595 (9)	0.0105 (7)	0.0164 (7)	0.0180 (7)
C3	0.0643 (9)	0.0473 (7)	0.0662 (9)	0.0058 (6)	0.0128 (7)	0.0236 (7)
C4	0.0552 (8)	0.0470 (7)	0.0594 (8)	0.0096 (6)	0.0120 (6)	0.0246 (6)
C5	0.0579 (8)	0.0468 (7)	0.0584 (8)	0.0077 (6)	0.0128 (6)	0.0224 (6)
C6	0.0648 (9)	0.0510 (8)	0.0658 (9)	0.0066 (7)	0.0203 (7)	0.0264 (7)
C7	0.0537 (8)	0.0492 (7)	0.0621 (8)	0.0076 (6)	0.0110 (6)	0.0282 (6)
C8	0.0634 (9)	0.0544 (8)	0.0630 (9)	0.0046 (7)	0.0191 (7)	0.0235 (7)
C16	0.0520 (8)	0.0596 (9)	0.0731 (10)	0.0040 (7)	0.0110 (7)	0.0357 (8)
C17	0.0671 (10)	0.0711 (10)	0.0861 (11)	0.0114 (8)	0.0254 (9)	0.0396 (9)
C18	0.0708 (11)	0.0944 (14)	0.1048 (14)	0.0091 (10)	0.0332 (10)	0.0577 (12)
C19	0.0731 (12)	0.0769 (12)	0.1180 (16)	−0.0086 (10)	0.0166 (11)	0.0561 (12)
C20	0.0793 (12)	0.0613 (10)	0.0965 (13)	−0.0043 (9)	0.0089 (10)	0.0351 (9)
C21	0.0682 (10)	0.0605 (9)	0.0770 (10)	0.0025 (8)	0.0145 (8)	0.0310 (8)
O4A	0.089 (5)	0.074 (4)	0.057 (3)	0.015 (2)	0.014 (2)	0.015 (2)
C9A	0.0606 (17)	0.0528 (15)	0.0582 (15)	0.0097 (13)	0.0137 (12)	0.0244 (12)
C10A	0.057 (2)	0.047 (2)	0.0600 (18)	0.0111 (16)	0.0153 (15)	0.0274 (15)
C11A	0.076 (3)	0.0520 (18)	0.068 (3)	0.0192 (18)	0.007 (2)	0.027 (2)
C12A	0.075 (3)	0.069 (3)	0.068 (3)	0.020 (2)	0.000 (3)	0.028 (2)
C13A	0.064 (3)	0.046 (2)	0.056 (3)	0.010 (2)	0.020 (3)	0.023 (2)
C14A	0.072 (3)	0.045 (2)	0.061 (2)	0.016 (2)	0.0172 (17)	0.0237 (17)
C15A	0.069 (2)	0.050 (2)	0.0616 (18)	0.0167 (16)	0.0118 (16)	0.0281 (17)
O4B	0.071 (3)	0.043 (3)	0.065 (4)	0.010 (2)	0.008 (2)	0.012 (2)
C9B	0.060 (2)	0.0465 (17)	0.0572 (19)	0.0071 (15)	0.0107 (15)	0.0258 (14)
C10B	0.056 (3)	0.044 (2)	0.057 (3)	0.0135 (18)	0.015 (2)	0.0265 (18)
C11B	0.080 (4)	0.053 (2)	0.065 (3)	0.019 (2)	0.008 (2)	0.027 (2)
C12B	0.074 (4)	0.054 (2)	0.058 (4)	0.016 (2)	0.001 (3)	0.020 (2)
C13B	0.069 (4)	0.051 (3)	0.052 (3)	0.007 (3)	0.025 (3)	0.017 (3)
C14B	0.086 (4)	0.049 (3)	0.078 (3)	0.025 (3)	0.022 (3)	0.029 (3)
C15B	0.074 (3)	0.052 (3)	0.066 (2)	0.020 (3)	0.009 (2)	0.030 (2)
O5A	0.096 (5)	0.144 (6)	0.142 (6)	0.022 (4)	0.043 (4)	0.105 (5)
C22A	0.240 (10)	0.303 (12)	0.320 (11)	0.152 (9)	0.161 (9)	0.258 (10)
C23A	0.192 (9)	0.210 (9)	0.186 (8)	0.048 (7)	0.103 (7)	0.160 (7)
O5B	0.083 (4)	0.072 (2)	0.075 (3)	0.003 (2)	0.018 (3)	0.029 (2)
C22B	0.144 (6)	0.257 (9)	0.089 (4)	−0.003 (6)	0.035 (4)	0.045 (5)
C23B	0.132 (6)	0.170 (7)	0.108 (5)	−0.040 (5)	0.033 (4)	0.019 (5)

### Geometric parameters (Å, º)

**Table:**

O1—H1	0.8200	C9A—C10A	1.505 (4)
O1—C1	1.3715 (18)	C10A—C11A	1.388 (5)
O2—H2	0.8200	C10A—C15A	1.373 (5)
O2—C3	1.3595 (18)	C11A—H11A	0.9300
O3—C5	1.3680 (17)	C11A—C12A	1.377 (5)
O3—C9A	1.395 (3)	C12A—H12A	0.9300
O3—C9B	1.380 (4)	C12A—C13A	1.368 (5)
N1—N2	1.3604 (18)	C13A—C14A	1.375 (6)
N1—C7	1.2953 (19)	C14A—H14A	0.9300
N2—H2A	0.8600	C14A—C15A	1.380 (5)
N2—C16	1.3896 (18)	C15A—H15A	0.9300
C1—C2	1.386 (2)	O4B—H4B	0.8200
C1—C6	1.381 (2)	O4B—C13B	1.374 (7)
C2—H2B	0.9300	C9B—H9B	0.9800
C2—C3	1.378 (2)	C9B—C10B	1.509 (5)
C3—C4	1.407 (2)	C10B—C11B	1.380 (6)
C4—C5	1.402 (2)	C10B—C15B	1.371 (6)
C4—C7	1.459 (2)	C11B—H11B	0.9300
C5—C6	1.382 (2)	C11B—C12B	1.365 (6)
C6—H6	0.9300	C12B—H12B	0.9300
C7—C8	1.495 (2)	C12B—C13B	1.363 (7)
C8—H8A	0.9700	C13B—C14B	1.372 (7)
C8—H8B	0.9700	C14B—H14B	0.9300
C8—H8BC	0.9700	C14B—C15B	1.383 (6)
C8—H8BD	0.9700	C15B—H15B	0.9300
C8—C9A	1.484 (3)	O5A—H5A	0.8200
C8—C9B	1.496 (4)	O5A—C23A	1.425 (7)
C16—C17	1.389 (2)	C22A—H22A	0.9600
C16—C21	1.389 (2)	C22A—H22B	0.9600
C17—H17	0.9300	C22A—H22C	0.9600
C17—C18	1.384 (2)	C22A—C23A	1.198 (13)
C18—H18	0.9300	C23A—H23A	0.9700
C18—C19	1.375 (3)	C23A—H23B	0.9700
C19—H19	0.9300	O5B—H5B	0.8200
C19—C20	1.368 (3)	O5B—C23B	1.462 (7)
C20—H20	0.9300	C22B—H22D	0.9600
C20—C21	1.390 (2)	C22B—H22E	0.9600
C21—H21	0.9300	C22B—H22F	0.9600
O4A—H4A	0.8200	C22B—C23B	1.237 (14)
O4A—C13A	1.376 (6)	C23B—H23C	0.9700
C9A—H9A	0.9800	C23B—H23D	0.9700
			
C1—O1—H1	109.5	C15A—C10A—C11A	118.4 (3)
C3—O2—H2	109.5	C10A—C11A—H11A	120.2
C5—O3—C9A	116.51 (15)	C12A—C11A—C10A	119.5 (4)
C5—O3—C9B	117.93 (16)	C12A—C11A—H11A	120.2
C7—N1—N2	118.79 (13)	C11A—C12A—H12A	119.1
N1—N2—H2A	119.9	C13A—C12A—C11A	121.8 (5)
N1—N2—C16	120.22 (13)	C13A—C12A—H12A	119.1
C16—N2—H2A	119.9	C12A—C13A—O4A	122.9 (8)
O1—C1—C2	117.22 (14)	C12A—C13A—C14A	118.8 (6)
O1—C1—C6	121.70 (14)	C14A—C13A—O4A	118.0 (8)
C6—C1—C2	121.08 (14)	C13A—C14A—H14A	120.1
C1—C2—H2B	120.2	C13A—C14A—C15A	119.8 (5)
C3—C2—C1	119.57 (14)	C15A—C14A—H14A	120.1
C3—C2—H2B	120.2	C10A—C15A—C14A	121.6 (4)
O2—C3—C2	117.53 (14)	C10A—C15A—H15A	119.2
O2—C3—C4	120.95 (14)	C14A—C15A—H15A	119.2
C2—C3—C4	121.52 (14)	C13B—O4B—H4B	109.5
C3—C4—C7	123.41 (13)	O3—C9B—C8	115.2 (3)
C5—C4—C3	116.68 (13)	O3—C9B—H9B	105.5
C5—C4—C7	119.91 (13)	O3—C9B—C10B	109.5 (3)
O3—C5—C4	121.29 (13)	C8—C9B—H9B	105.5
O3—C5—C6	116.16 (13)	C8—C9B—C10B	114.6 (3)
C6—C5—C4	122.55 (13)	C10B—C9B—H9B	105.5
C1—C6—C5	118.60 (14)	C11B—C10B—C9B	119.1 (5)
C1—C6—H6	120.7	C15B—C10B—C9B	122.3 (5)
C5—C6—H6	120.7	C15B—C10B—C11B	118.6 (5)
N1—C7—C4	118.58 (13)	C10B—C11B—H11B	119.4
N1—C7—C8	124.66 (14)	C12B—C11B—C10B	121.1 (6)
C4—C7—C8	116.75 (12)	C12B—C11B—H11B	119.4
C7—C8—H8A	108.8	C11B—C12B—H12B	119.9
C7—C8—H8B	108.8	C13B—C12B—C11B	120.1 (7)
C7—C8—H8BC	109.3	C13B—C12B—H12B	119.9
C7—C8—H8BD	109.3	C12B—C13B—O4B	113.9 (10)
C7—C8—C9B	111.81 (17)	C12B—C13B—C14B	119.6 (7)
H8A—C8—H8B	107.7	C14B—C13B—O4B	125.5 (11)
H8BC—C8—H8BD	107.9	C13B—C14B—H14B	119.9
C9A—C8—C7	113.85 (15)	C13B—C14B—C15B	120.2 (7)
C9A—C8—H8A	108.8	C15B—C14B—H14B	119.9
C9A—C8—H8B	108.8	C10B—C15B—C14B	120.2 (6)
C9B—C8—H8BC	109.3	C10B—C15B—H15B	119.9
C9B—C8—H8BD	109.3	C14B—C15B—H15B	119.9
C17—C16—N2	117.90 (15)	C23A—O5A—H5A	109.5
C17—C16—C21	119.15 (15)	H22A—C22A—H22B	109.5
C21—C16—N2	122.94 (15)	H22A—C22A—H22C	109.5
C16—C17—H17	120.0	H22B—C22A—H22C	109.5
C18—C17—C16	120.09 (18)	C23A—C22A—H22A	109.5
C18—C17—H17	120.0	C23A—C22A—H22B	109.5
C17—C18—H18	119.5	C23A—C22A—H22C	109.5
C19—C18—C17	120.91 (18)	O5A—C23A—H23A	104.5
C19—C18—H18	119.5	O5A—C23A—H23B	104.5
C18—C19—H19	120.5	C22A—C23A—O5A	130.9 (14)
C20—C19—C18	118.93 (17)	C22A—C23A—H23A	104.5
C20—C19—H19	120.5	C22A—C23A—H23B	104.5
C19—C20—H20	119.2	H23A—C23A—H23B	105.7
C19—C20—C21	121.52 (19)	C23B—O5B—H5B	109.5
C21—C20—H20	119.2	H22D—C22B—H22E	109.5
C16—C21—C20	119.38 (17)	H22D—C22B—H22F	109.5
C16—C21—H21	120.3	H22E—C22B—H22F	109.5
C20—C21—H21	120.3	C23B—C22B—H22D	109.5
C13A—O4A—H4A	109.5	C23B—C22B—H22E	109.5
O3—C9A—C8	115.0 (2)	C23B—C22B—H22F	109.5
O3—C9A—H9A	106.1	O5B—C23B—H23C	108.1
O3—C9A—C10A	108.3 (2)	O5B—C23B—H23D	108.1
C8—C9A—H9A	106.1	C22B—C23B—O5B	116.8 (12)
C8—C9A—C10A	114.4 (2)	C22B—C23B—H23C	108.1
C10A—C9A—H9A	106.1	C22B—C23B—H23D	108.1
C11A—C10A—C9A	121.1 (4)	H23C—C23B—H23D	107.3
C15A—C10A—C9A	120.5 (4)		
			
O1—C1—C2—C3	178.29 (15)	C7—C8—C9A—O3	−42.0 (3)
O1—C1—C6—C5	−178.82 (15)	C7—C8—C9A—C10A	−168.4 (3)
O2—C3—C4—C5	−179.31 (15)	C7—C8—C9B—O3	46.7 (4)
O2—C3—C4—C7	0.1 (3)	C7—C8—C9B—C10B	175.3 (3)
O3—C5—C6—C1	−179.85 (14)	C8—C9A—C10A—C11A	64.1 (5)
O3—C9A—C10A—C11A	−65.7 (5)	C8—C9A—C10A—C15A	−116.3 (4)
O3—C9A—C10A—C15A	113.9 (4)	C8—C9B—C10B—C11B	107.9 (6)
O3—C9B—C10B—C11B	−120.8 (6)	C8—C9B—C10B—C15B	−70.3 (6)
O3—C9B—C10B—C15B	61.0 (6)	C16—C17—C18—C19	0.4 (3)
N1—N2—C16—C17	−175.73 (15)	C17—C16—C21—C20	0.0 (3)
N1—N2—C16—C21	4.2 (3)	C17—C18—C19—C20	−0.5 (3)
N1—C7—C8—C9A	−164.3 (2)	C18—C19—C20—C21	0.3 (3)
N1—C7—C8—C9B	153.3 (2)	C19—C20—C21—C16	0.0 (3)
N2—N1—C7—C4	−178.82 (13)	C21—C16—C17—C18	−0.2 (3)
N2—N1—C7—C8	1.6 (2)	O4A—C13A—C14A—C15A	−177.1 (16)
N2—C16—C17—C18	179.73 (17)	C9A—O3—C5—C4	−26.8 (3)
N2—C16—C21—C20	−179.91 (17)	C9A—O3—C5—C6	153.5 (2)
C1—C2—C3—O2	−179.82 (15)	C9A—C10A—C11A—C12A	−179.9 (7)
C1—C2—C3—C4	0.7 (3)	C9A—C10A—C15A—C14A	−179.2 (6)
C2—C1—C6—C5	0.4 (3)	C10A—C11A—C12A—C13A	−2.8 (18)
C2—C3—C4—C5	0.2 (2)	C11A—C10A—C15A—C14A	0.4 (9)
C2—C3—C4—C7	179.60 (15)	C11A—C12A—C13A—O4A	177.8 (18)
C3—C4—C5—O3	179.56 (14)	C11A—C12A—C13A—C14A	4 (3)
C3—C4—C5—C6	−0.8 (2)	C12A—C13A—C14A—C15A	−3 (3)
C3—C4—C7—N1	5.3 (2)	C13A—C14A—C15A—C10A	0.8 (18)
C3—C4—C7—C8	−175.16 (15)	C15A—C10A—C11A—C12A	0.6 (9)
C4—C5—C6—C1	0.5 (3)	O4B—C13B—C14B—C15B	170 (2)
C4—C7—C8—C9A	16.1 (3)	C9B—O3—C5—C4	20.7 (3)
C4—C7—C8—C9B	−26.2 (3)	C9B—O3—C5—C6	−159.0 (3)
C5—O3—C9A—C8	48.0 (3)	C9B—C10B—C11B—C12B	178.9 (9)
C5—O3—C9A—C10A	177.4 (2)	C9B—C10B—C15B—C14B	−179.8 (9)
C5—O3—C9B—C8	−44.8 (4)	C10B—C11B—C12B—C13B	3 (2)
C5—O3—C9B—C10B	−175.9 (3)	C11B—C10B—C15B—C14B	1.9 (12)
C5—C4—C7—N1	−175.36 (14)	C11B—C12B—C13B—O4B	−172 (2)
C5—C4—C7—C8	4.2 (2)	C11B—C12B—C13B—C14B	−3 (4)
C6—C1—C2—C3	−1.0 (3)	C12B—C13B—C14B—C15B	2 (4)
C7—N1—N2—C16	−172.39 (14)	C13B—C14B—C15B—C10B	−2 (2)
C7—C4—C5—O3	0.1 (2)	C15B—C10B—C11B—C12B	−2.8 (12)
C7—C4—C5—C6	179.81 (14)		

### Hydrogen-bond geometry (Å, º)

**Table:**

*D*—H···*A*	*D*—H	H···*A*	*D*···*A*	*D*—H···*A*
O5*A*—H5*A*···C17	0.82	2.56	3.363 (15)	166
O5*A*—H5*A*···C18	0.82	2.47	3.263 (16)	162
O5*B*—H5*B*···C19^i^	0.82	2.59	3.405 (11)	173
O1—H1···O5*A*^i^	0.82	1.79	2.590 (12)	166
O1—H1···O5*B*	0.82	1.90	2.709 (8)	170
C8—H8*BC*···O4*B*^ii^	0.97	2.49	3.440 (17)	168
O4*A*—H4*A*···O1^iii^	0.82	1.89	2.677 (13)	160
C9*B*—H9*B*···O2^i^	0.98	2.39	3.347 (5)	165
O2—H2···N1	0.82	1.87	2.5975 (18)	147

Symmetry codes: (i) −*x*+1, −*y*+1, −*z*+1; (ii) −*x*+1, −*y*+2, −*z*+2; (iii) *x*, *y*+1, *z*+1.
